# Two jasmonic acid carboxyl methyltransferases in *Gossypium hirsutum* involved in MeJA biosynthesis may contribute to plant defense

**DOI:** 10.3389/fpls.2023.1249226

**Published:** 2023-09-05

**Authors:** Dong Teng, Weixia Jing, Beibei Lv, Xinzheng Huang, Danyang Zhao, Junfeng Kou, Xiaohe Liu, Khalid Hussain Dhiloo, Yongjun Zhang

**Affiliations:** ^1^ State Key Laboratory for Biology of Plant Diseases and Insect Pests, Institute of Plant Protection, Chinese Academy of Agricultural Sciences, Beijing, China; ^2^ Key Laboratory of Integrated Management of Crop Diseases and Pests (Ministry of Education), College of Plant Protection, Nanjing Agricultural University, Nanjing, China; ^3^ College of Plant Protection, Shandong Agricultural University, Taian, China; ^4^ College of Plant Protection, China Agricultural University, Beijing, China; ^5^ School of Resources and Environment, Henan Institute of Science and Technology, Xinxiang, China; ^6^ Institute of Plant Protection, Cangzhou Academy of Agriculture and Forestry Sciences, Cangzhou, China; ^7^ Department of Entomology, Faculty of Crop Protection, Sindh Agriculture University, Tandojam, Pakistan

**Keywords:** phytohormone, jasmonic acid carboxyl methyltransferases, biosynthesis, HIPVs, plant defense

## Abstract

Jasmonic acid (JA) and methyl jasmonate (MeJA), the crucial plant hormones, can induce the emission of plant volatiles and regulate the behavioral responses of insect pests or their natural enemies. In this study, two jasmonic acid carboxyl methyltransferases (JMTs), GhJMT1 and GhJMT2, involved in MeJA biosynthesis in *Gossypium. hirsutum* were identified and further functionally confirmed. *In vitro*, recombinant GhJMT1 and GhJMT2 were both responsible for the conversion of JA to MeJA. Quantitative real-time PCR (qPCR) measurement indicated that *GhJMT1* and *GhJMT2* were obviously up-regulated in leaves and stems of *G. hirsutum* after being treated with MeJA. In gas chromatography-mass spectrometry (GC-MS) analysis, MeJA treatment significantly induced plant volatiles emission such as (*E*)-β-ocimene, (*Z*)-3-hexenyl acetate, linalool and (3*E*)-4,8-dimethyl-1,3,7-nonatriene (DMNT), which play vital roles in direct and indirect plant defenses. Moreover, antennae of parasitoid wasps *Microplitis mediator* showed electrophysiological responses to MeJA, β-ocimene, *(Z)*-3-hexenyl acetate and linalool at a dose dependent manner, while our previous research revealed that DMNT excites electrophysiological responses and behavioral tendencies. These findings provide a better understanding of MeJA biosynthesis and defense regulation in upland cotton, which lay a foundation to JA and MeJA employment in agricultural pest control.

## Introduction

In nature, plants are very often attacked by massive pests and abiotic factors. Thereupon, plants have evolved a sophisticated defense system including direct defenses (accumulation of toxic chemicals) and indirect defenses (releasing semiochemicals to attract natural enemies) against herbivore infestation (Howe and Jander, 2008; Turlings and Erb, 2018). In response to insect attack, plants release complex mixure of volatiles (Held et al., 2010; Degenhardt et al., 2011). Upon herbivore infestation, the herbivore induced plant volatiles (HIPVs) are released, which undertake two main roles in plant defense: 1) attracting or repelling conspecifics of the herbivores, and 2) attracting natural enemies of hebivores. Further, plants releasing HIPVs alert nearby plants and improve their own defenses (Dicke and van Loon, 2000).

Jasmonic acid (JA) and methyl jasmonate (MeJA), produced by many plants, are naturally occurring compounds (Chen et al., 2022). As major phytohormones, JA and MeJA contribute to plant growth, plant development and plant defense (Baldwin et al., 2002). More and more investigations are focusing on the defense roles of MeJA and JA in many plants against insects (Baldwin et al., 2002; Bostock, 2005). Exogenous MeJA treatments induce plant defense against herbivores in widely plant taxa (Heijari et al., 2005; Erbilgin et al., 2006; Tan et al., 2011). For instance, the population growth of green peach aphids feeding on tomato plants treatd by MeJA were significantly suppressed (Boughton et al., 2006). Similarly, on soybean plants pretreated with MeJA, population of both soybean thrips and soybean aphids were downgraged 47 and 25%, respectively (Selig et al., 2016). When *Manduca sexta* larvae were confined to MeJA-induced *Nicotiana attenuata* plant leaves, they experienced higher mortality rates, grew slower and attained lower body masses than those fed only control leaves (Dam et al., 2000). In addition, there were dramatic reduction in relative growth rates of *M. sexta* larvae feeding on *N. attenuata* plants induced by 250 μg of MeJA (Pohlon and Baldwin, 2003). Most induced responses in plants display a negative impact on insect growth and development. Nevertheless, application of exogenous MeJA on transgenic cotton induces cotton plant responses, but not results in concomitant responses by insects (Williams et al., 2017).

The methylation of MeJA and Methyl salicylate (MeSA) are catalysed by a special class of enzymes, most of them are JA methyltransferase (JMT) and salicylic acid (SA) methyltransferase (SAMT) within the same family called SABATH (Zhao et al., 2009). The SABATH family specifically catalyses the methylation of carboxylic acids and nitrogen atoms (Zhao et al., 2013). Moreover, SABATH family proteins catalyse *S*-adenosyl-L-methionine (SAM)-dependent methylation of hormones, signal molecules and other metabolites in plants (D’Auria et al., 2003). Recently, SABATH family has been discovered in a varity of plant species. There are 24 SABATHs identified in *Arabidopsis thalian*a, 28 SABATHs in *Populus trichocarpa*, 30 SABATHs in *Salvia miltiorrhiza* and 41 SABATHs in rice *Oryza sativa* (Zhao et al., 2013; Wang et al., 2017).

Cotton plants suffer from infestation by a wide range of destructive insect pests. However, little is known about the detailed roles of JA and MeJA in cotton plants defense. In this study, we newly identified two jasmonic acid carboxyl methyltransferase genes (*GhJMT1* and *GhJMT2*) in *Gossypium hirsutum*. The catalytic functions of recombiant GhJMT1 and GhJMT2 were characterized by performing *in vitro* enzyme assays. Subsequently, quantitative real-time PCR (qPCR) was conducted to determine the expression levels of *GhJMT1* and *GhJMT2* in cotton plants after MeJA induction. Using dynamic headspace sampling system coupled with gas chromatography-mass spectrometer (GC-MS) analysis, cotton volatile blends induced by MeJA were measured. Further, we also investigated the electroantennogram (EAG) responses of parasitoid wasp *Microplitis mediator* to MeJA and its induced volatile compounds. These findings will provide valuable insights in biosynthesis and regulated roles of MeJA in cotton and contribute to utilizing MeJA and its analogues in plant defense.

## Materials and methods

### Plant and insect material

Cotton seeds (*G. hirsutum* L. cv. Zhongmian 12) were sown in plastic pots (height, 14 cm; diameter, 16 cm) and placed in a greenhouse. Cotton plants were irrigated every two days. The cocoons of *M. mediator* were kindly provided by Plant Protection Institute, Hebei Academy of Agricultural and Forestry Sciences, China. Parasitoid cocoons were reared in an artificial climate incubator with a condition of 28 ± 1°C, 60 ± 10% R.H. (Relative Humidity) and 16L: 8D photoperiod. The wasp adults after eclosion were fed with a 10% sucrose solution. One to two days old adults were prepared for the next experiments.

### MeJA induction

In MeJA treatment, one cotton plant with 6−7 fully expanded leaves was placed into a glass jar (10 cm in diameter × 25 cm in height). Plant was treated with a cotton ball containing 40 μL of standard ethanol-MeJA (9:1) solution. Two cotton balls were placed underneath cotton leaves, without physical contact with the treated plants. Hormone induction lasted for 18 h, starting at 6:00 pm. Control plant was treated with cotton balls only containing the pure ethanol. After induction, top three leaves, middle three leaves, bottom three leaves, stems and roots were collected to determine the expression profiles of two target genes by performing qPCR measurement, and parallel volatile collections were conducted according to the following description. Each treatment was repeated six times.

### Target GhJMTs identification and expression analysis

The protein sequence of *A. thaliana* AtJMT (Accession number: AY008434) was used initially as a query sequence to search against the *G. hirsutum* database using BLASTN program. Amino acid sequences of candidate JMTs were aligned using CLUSTALW (https://www.genome.jp/tools-bin/clustalw). Based on the amino acid sequences of JMTs, a neighbor-joining phylogenetic tree was constructed using MEGA 11.0.

The RNAprep Pure Plant Kit (TIANGEN, Beijing, China) was used to extract total RNA from cotton tissue sample. RNA quality was evaluated using 1.5% agarose gel electrophoresis analysis and a NanoDrop 2000c spectrophotometer (NanoDrop, Wilmington, DE). The FastQuant RT Kit (TIANGEN, Beijing, China) was utilized to synthesize cDNA, then the cDNA was stored for further use.

The qPCR measurement was carried out to determine the expression of *GhJMT1* and *GhJMT2* in cotton plants induced by MeJA. The *GhACT4* (Accession number: AY305726) was applied as a reference gene (Artico et al., 2010). Specific primers for qPCR were designed using Beacon Designer™ 8.0 (**Table 1**). Each PCR reaction was conducted in a total volume of 20 μL mixture containing 1μL of the template cDNA, 2×SuperReal PreMix Plus (SYBR), 10 µM of each primer, and 50×ROX Reference Dye. All PCRs were performed on an ABI Prism 7500 Fast Detection System (Applied Biosystems, CA, USA) with the following cycle conditions: 95°C for 10 min followed by 40 cycles of 95°C for 10s, 60°C 32s. The comparative 2^-ΔΔCT^ method was employed to calculate the relative transcript levels of target genens (Livak and Schmittgen, 2001).

**Table 1:** Specific primers used in gene cloning and qPCR.

Primers	sequence (5’–3’)
For gene cloning	
JMT1-F	ATGCAAGTACTTCACATGAACA
JMT1-R	TTACCACTTTTTAGTGAGGGC
JMT2-F	ATGGAAGTAGTGCAAGTGCTTCA
JMT2-R	TCAAAAAAGAAAAACATCCATAGG
For qPCR	
JMT1-F	TCAAAGAGCAGCCCACAGAG
JMT1-R	CCGGCCCATGAAAGAAAGGA
JMT2-F	GGAAAGCTATGCCGTGAAGC
JMT2-R	GCCCAGGAAAGAAAGGACCA
GhACT4-F	TGCAGACCGTATGAGCAAGG
GhACT4-R	GCTGGAAGGTGCTGAGTGAT

### Heterologous expression and catalytic assay

Gene-specific primers were designed to clone the full-length cDNAs of JMTs using Beacon Designer 7.9 (**Table 1**). The target gene were cloned into the expression vector pET28a (+) (Biomed, Beijing, China). Then, plasmid containing the correct insert was transformed into the *E. coli* strain BL21 (DE3) for heterologous expression. Freshly transformed *Escherichia coli* cells were cultured in 500 mL LB medium with 50 μg/mL of kanamycin at 37°C, vibration of 220 rpm till to an OD_600_ of 0.6. Then, 1 mM isopropyl 1-thio-β-D-galactopyranoside (IPTG) was added, and the cultures were vibrated at 150 rpm at 16°C for 20 h. The crude protein was pelleted by centrifugation of 8500 × *g* at 4°C for 30 min and then resuspended in 30 mL lysis buffer (100 mM NaCl, 0.5% TritionX-100, 50 mM Tris-Hcl, 2 mM EDTA, pH 8.5), added 150 μL 1 M of dithiothreitol (DTT) and 3 μL 100 mM of phenylmethanesulfonyl fluoride (PMSF). After sonication, the suspension was centrifuged under 16000 × *g* at 4°C for 30 min, and finally protein supernatant was harvested.

Enzyme catalysis assays were performed in 20-mL PTFE/Silicon Septa screw cap glass vials (Agilent Technologies, USA) using JA, SA, 4-hydroxy-benzoic acid and *trans*-cinnamic acid as substrate, respectively. All substrates were standard compounds. In a vial, added 88 μL of recombinant protein supernatant, 10 μL 1 M of KCl, 1 μL 100 mM of SAM, 1 μL of JA or SA or 4-hydroxy-benzoic acid or *trans*-cinnamic acid, separately. Reaction samples were incubated at 25°C for 30 min, then added 300 µL of ethyl acetate, vibration of 150 rpm at 20°C for 10min. The organic phase was gathered and a sample volume of 1 μL was injected in a Shimadzu GC-MS (GC-MS-QP2010 SE, Japan) on an Rxi-5Sil MS column (30 m × 0.250 mm × 0.25 μm, Restek, PA, USA). The GC oven temperature program was 100°C for 2 min followed by an increase to 170°C at a rate of 10°C/min (2-min hold) and then to 280°C at a rate of 5°C/min (5-min hold). Other parameters were: injector temperature 250°C; ion source temperature 250°C; injector and ion source temperature 250°C; EI 70 eV; carrier gas (helium) at a flow rate of 1 ml/min; mass range 50-650 m/z. Products were identified by comparison of their retention times and mass spectra with those of authentic standards (Sigma‐Aldrich) analysed under the same conditions.

### Plant volatiles collection and determination

To collect cotton plant volatiles, dynamic headspace sampling method was carried out. One MeJA-exposed or control plant in pot were randomly placed within a glass jar at 18 h after onset of MeJA. The container was sealed with a glass lid that had an air inlet and an air outlet. Air was purified by passage through an activated charcoal filter and pumped through the container at a flow rate of 1500 mL/min with a vacuum pump (Beijing Institute of Labor Instrument, Beijing, China). Eight mm diameter glass tubes comtaining 50 mg of 60/80 mesh Tenax TA (Shanghai ANPEL Science Instrument Company, Shanghai, China) directly connected to the outlet were used to collect volatiles. The collection of volatiles for each treatment was repeated 3 times.

The collected volatiles were extracted with 300 μL of hexane (Fisher, Fairlawn, NJ). 8.65 ng/μL of ethyl caprate (Sigma-Aldrich, Oakville, Canada) was individually added in each extracted sample as an internal standard. Except for the following settings: the GC oven temperature program was 40°C (1-min hold) followed by a rise to 130°C at a rate of 4°C/min (5-min hold) and then to 250°C at a rate of 10°C/min (5-min hold), the rest of GC-MS analysis was executed as mentioned above.

### EAG recordings

Our previous research results revealed that (3*E*)-4,8-dimethyl-1,3,7-nonatriene (DMNT) excites electrophysiological responses and behavioral tendencies. Here, EAG recordings were utilized to assessed the electrophysiological responses ofantennae of *M. mediator* to MeJA, β-ocimene, (*Z*)-3-hexenyl acetate and linalool. Four standard compounds were diluted with mineral oil to a concentration of 0.001, 0.01, 0.1, 1, 10 and 100 μg/μL, separately. Mineral oil was used as the blank control, and *cis*-3-hexen-1-ol was used as the reference compound. Antenna from 3-day-old adult (males and females) was cautiously cut off from the base, and a few terminal segments at the distal end were removed, then the treated antenna was connected to electrode holders with electrode gel. Twenty microliter of odor solution was applied on a piece of folded filter paper (0.5×5 cm) and placed into a glass Pasteur pipette. A constant charcoal-filtered humid air flow (300 mL/min) through a metal tube was applied to each antenna for 0.5 s. Each compound was tested on 8 antennae. EAG signals were recorded and analyzed using Syntech IDAC-2 (Intelligent Data Acquisition Controller) and EAGPro V2.0 (Syntech, Kirchzarten, Germany), respectively. EAG data were calculated in the formula: EAG relative value = (EAG value of compound-EAG value of control)/(EAG value of reference-EAG value of control)×100.

### Statistical analysis

All data were analyzed by SPSS STATISTICS 18.0 software (SPSS Inc., Chicago, IL, USA). Data are presented as the mean ± standard error (SE) and if needed, were transformed prior to analysis. The differences of the volatile emission between control and treatment groups, comparisons of EAG values between two sexes, and the comparisons of target genes expression between control and treatment groups were assessed using paired-sample *t*-test (*P* < 0.05).

## Results

### Identification of GhJMTs in cotton

**Figure 1:**
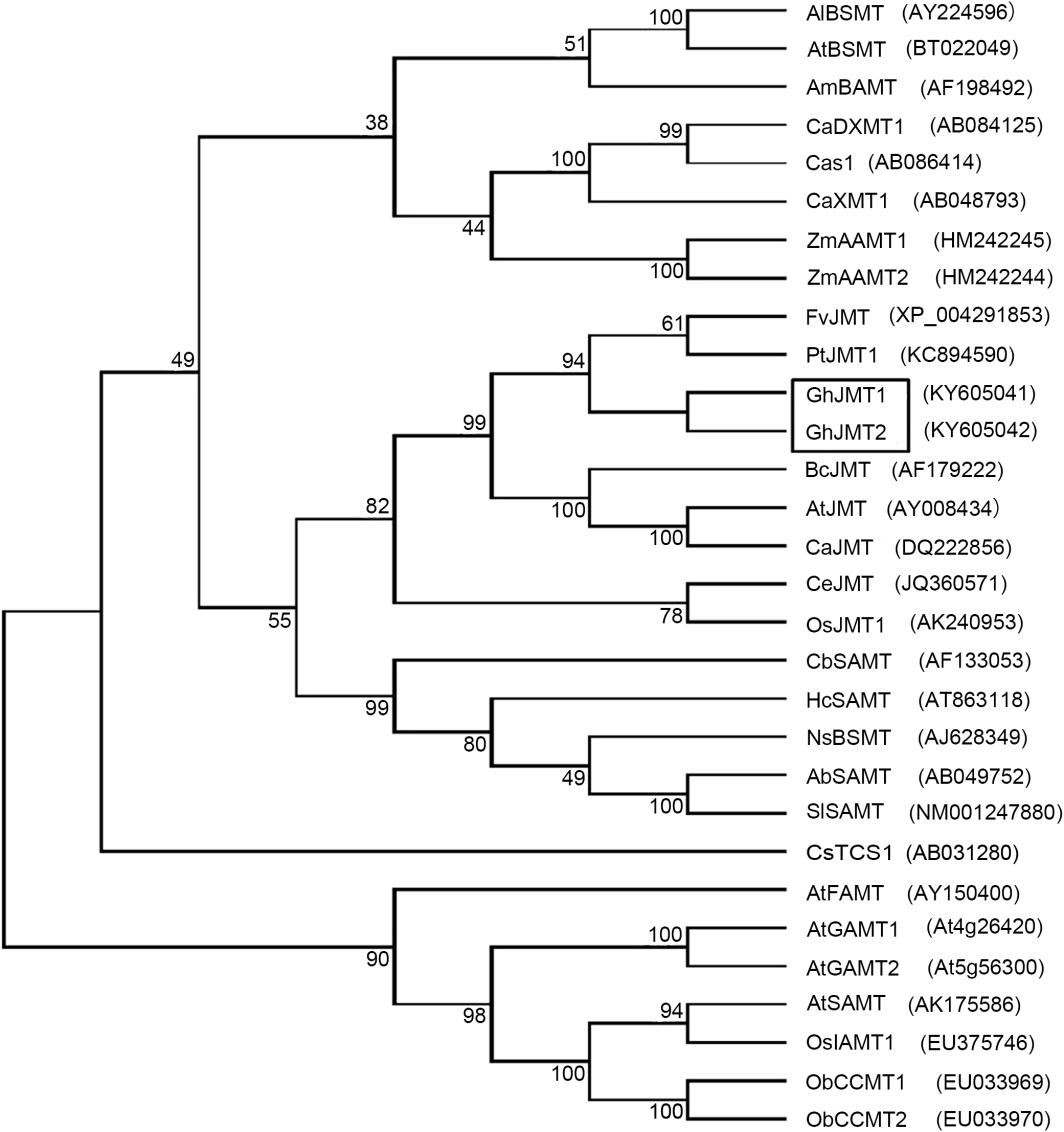
Phylogenetic tree analysis of two GhJMTs and other methylferases. Al, *Arabidopsis lyrata*; At, Arabidopsis thaliana; Am, Antirrhinum majus; Ca, Coffea canephora; Zm, Zea mays; Fv, Fragaria vesca; Pt, Populus trichocarpa; Gh, Gossypium hirsutum; Bc, Brassica campestris; Ce, Cymbidium ensifolium; Os, Oryza sativa; Cb, Clarkia breweri; Hc, Hoya carnosa; Ns, Nicotiana suaveolens; Ab, Atropa belladonna; Sl, Solanum lycopersicum; Ob, Ocimum basilicum.

To identify the putative cotton *JMTs*, the *AtJMT* sequence was used to blast search the *G. hirsutum* genome. The full-length cDNAs of *GhJMT1* (Genbank accession number: KY605041) and *GhJMT2* (Genbank accession number: KY605042) were obtained by the RT-PCR. The cDNA sequences of PCR products were1116bp and 1119bp, encoding predicted proteins of 372 and 373 amino acids, with predicted molecular masses of 41.5 and 41.7 kD, respectively. GhJMT1 (shared 57%−65% identity with those JMTs from strawberry, *Arabidopsis* and black cottonwood, while GhJMT2 shared 54%−64% identity. Additionally, GhJMT1 and GhJMT2 shared 72.7% identity. Phylogenetic analysis showed that GhJMT1 and GhJMT2 cluster in a clade containing antother jasmonic carboxyl methyltransferases of AtJMT, CeJMT, BcJMT, FvJMT, OsJMT1, CaJMT, PtJMT and NTR1 (**Figure 1**).

### Catalytic function of recombinant GhJMTs

**Figure 2:**
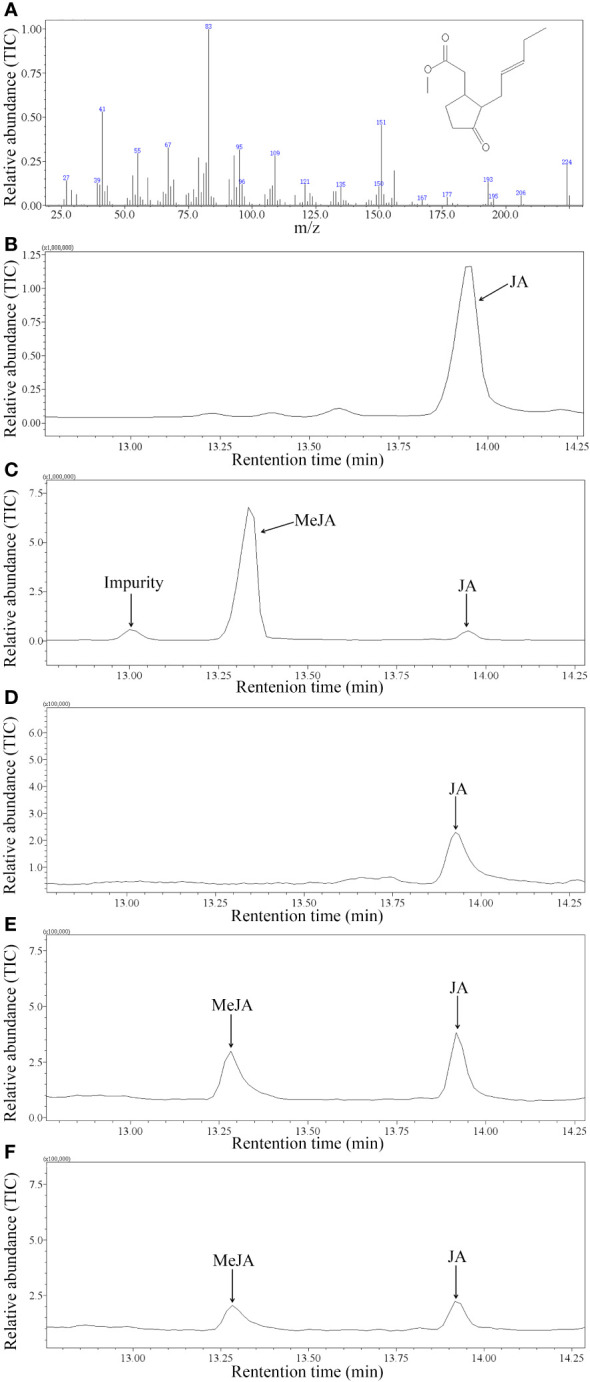
GC-MS analysis of products catalyzed by recombinant GhJMT1 and GhJMT2. **(A)** the mass spectrum of MeJA standard. **(B)** gas chromatogram of JA standard. **(C)** gas chromatogram of MeJA standard. **(D)** product catalyzed by the empty pET28a vector. **(E)** product catalyzed by GhJMT1; **(F)** product catalyzed by GhJMT2.

Based on mass spectra libraries (NIST and Department of Chemical Ecology, Gothenburg University, Sweden) together with the GC retention times and mass spectra of authentic standards, it was found that recombinant GhJMT1 and GhJMT2 were both responsible for the conversion of JA to MeJA (**Figure 2**). However, recombinant GhJMT1 and GhJMT2 had no catalytic abilities against other three substrates, SA, 4-hydroxy-benzoic acid and *trans*-cinnamic acid.

### Transcript abundance of *GhJMTs* in *G. hirsutum* induced by MeJA

**Figure 3:**
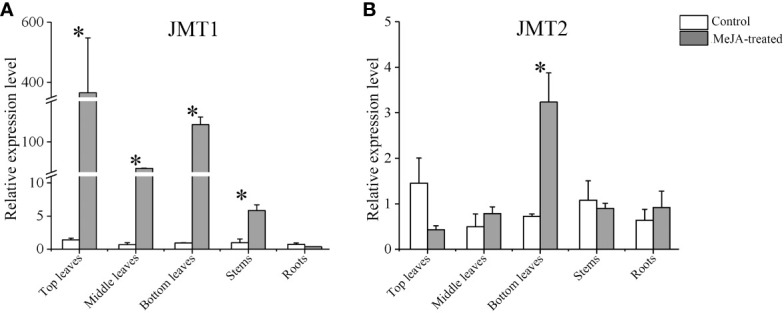
The *GhJMT1* and *GhJMT2* expressions in different tissues of cotton treated with MeJA. **(A)** The relative expression of *GhJMT1* induced by MeJA. **(B)** The relative expression of *GhJMT2* induced by MeJA. The asterisk indicates significant difference between treatment and control groups (*P* < 0.05).

The qPCR measurement was conducted to investigate the expression of *GhJMT1* and *GhJMT2* in MeJA-induced cotton plants. The results indicated that *GhJMT1* and *GhJMT2* were expressed in leaves, stems and roots of cotton plants. After MeJA treatment, the expression of *GhJMT1* was significantly increased in middle leaves, bottom leaves and stems, whereas the expression of *GhJMT2* was significantly increased in bottom leaves (**Figure 3**).

### Volatile emission from MeJA induced cotton plants

**Figure 4:**
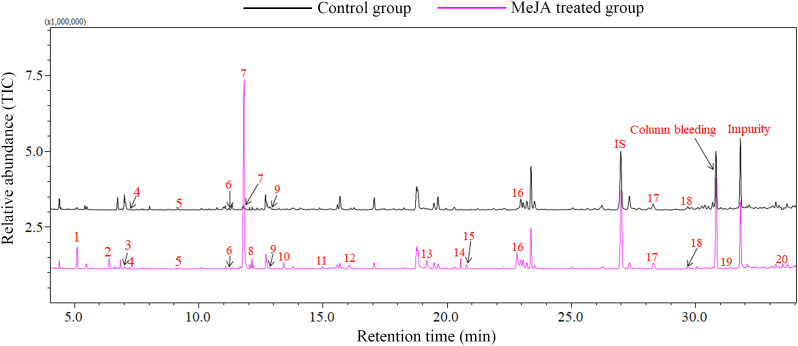
Volatile compounds emitted from MeJA treated and control cotton plants. 1, Butanoic acid, ethyl ester; 2, (*E*)-3-Hexen-1-ol; 3, (*E*)-2-Hexen-1-ol; 4, 3-Methyl butyl acetate; 5, α-Pinene; 6, β-Myrcene; 7, *c*is-3-Hexenyl acetate; 8, (*Z*)-2-Hexenyl acetate; 9, 1-Decyne; 10, β-Ocimene; 11, Linalool; 12, DMNT; 13, (*E*)-2-Hexenyl butyrate; 14, (*Z*)-3-Hexenyl 2-methylbutanoate; 15, (*Z*)-1-Ethoxy-4-methyl-2-pentene; 16, Indole; 17, β-Caryophyllene; 18, α-Humulene; 19, α-Farnesene; 20, (*E,E*)-4,8,12-Trimethyl-1,3,7,11-tridecatetraene. IS, internal standard; Column bleeding, Tetradecamethylcycloheptasiloxane.

In GC-MS analysis, MeJA treatment significantly induced the emission of plant volatiles. Approximately 20 volatile compounds were induced in MeJA treated cotton plants. Seventeen compounds including DMNT, (*E*)-β-ocimene, (*Z*)-3-hexenyl acetate and linalool were significantly up-regulated in MeJA treated cotton plants, in particular, 12 compounds were exclusively emitted from MeJA induced cotton plants, which play a pivotal impart in direct and indirect plant defenses. However, only the emssion of β-myrcene was down-regulated in MeJA treated cotton plants (**Figure 4** **;** **Table 2**).

**Table 2:** Proportion (% of internal standard) of volatile compounds emitted from cotton plants induced by MeJA.

Number	Compounds	Control groups	MeJA treated groups
1	Butanoic acid, ethyl ester	N.D.	26.1 ± 11.31 * ↑
2	(*E*)-3-Hexen-1-ol	N.D.	14.93 ± 12.76 * ↑
3	(*E*)-2-Hexen-1-ol	N.D.	1.92 ± 1.29 * ↑
4	3-Methyl butyl acetate	0.23 ± 0.04	4.6 ± 1.53 * ↑
5	α-Pinene	1.39 ± 0.29	1.83 ± 0.52 ^ns^ −
6	β-Myrcene	1.41 ± 0.46 *	0.42 ± 0.08 ↓
7	*c*is-3-Hexenyl acetate	1.37 ± 0.36	139.08 ± 40.5 * ↑
8	(*Z*)-2-Hexenyl acetate	N.D.	7.84 ± 1.24 * ↑
9	1-Decyne	1.53 ± 0.75	1.43 ± 0.78 ^ns^ −
10	β-Ocimene	N.D.	5.85 ± 0.29 * ↑
11	Linalool	N.D.	2.7 ± 0.29 * ↑
12	DMNT	N.D.	5.73 ± 0.33 * ↑
13	(*E*)-2-Hexenyl butyrate	N.D.	6.04 ± 3.03 * ↑
14	(*Z*)-3-Hexenyl 2-methylbutanoate	N.D.	7.93 ± 3.06 * ↑
15	(*Z*)-1-Ethoxy-4-methyl-2-pentene	N.D.	9.45 ± 2.69 * ↑
16	Indole	9.65 ± 4.46	20.37 ± 6.33 * ↑
17	β-Caryophyllene	12.04 ± 2.95	9.29 ± 4.14 ^ns^ −
18	α-Humulene	4.92 ± 1.46	2.73 ± 1.3 ^ns^ −
19	α-Farnesene	N.D.	3.86 ± 2.72 * ↑
20	(*E,E*)-4,8,12-Trimethyl-1,3,7,11-tridecatetraene (TMTT)	N.D.	2.42 ± 0.44 * ↑

N.D., not detected. Data in the same line followed by asterisk are significantly different (P<0.05). ↑ indicates up-regulated. ↓ indicates down-regulated. The ns and − indicate no significant difference.

### Electrophysiological response

**Figure 5:**
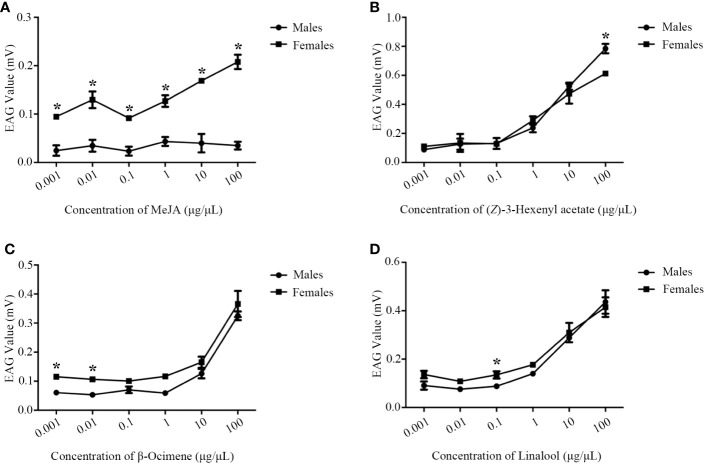
The dose-dependent manner of *M. mediator* antennae to MeJA **(A)**, β-ocimene **(B)**, (*Z*)-3-hexenyl acetate **(C)** and linalool **(D)**. The asterisk indicates significant difference (*P* < 0.05).

EAGs of *M. mediator* to MeJA, β-ocimene, (*Z*)-3-hexenyl acetate and linalool showed a dose-dependent manner (**Figure 5**). The EAG values of *M. mediator* females to MeJA were higher than those of males (**Figure 5A**). There were no statistical differences in EAG responses to β-ocimene, (*Z*)-3-hexenyl acetate or linalool between male and female *M. mediator* (**Figures 5B–D**).

## Discussion

In plants, indole-3-acetic acid (IAA), gibberellins (GAs), SA and JA as important plant signaling molecules are usually in the form of methyl esters (Westfall et al., 2013). IAA methyltransferase (IAMT), GA methyltransferase (GAMT), SAMT and JMT belonging to “SABATH” family are separately responsible for catalyzing the methylation of IAA, GA, SA, and JA (D’Auria et al., 2003). Lots of SABATH methyltransferases are identified in *Arabidopsis* and rice (Zhao et al., 2008). SABATH methyltransferases often conduce to the formation of unique scents of plants appealing to animals, and also are involved in the regulation of plants’ diverse developmental processes (Creelman and Mullet, 1997; Wasternack and Hause, 2002). JMT is a critical enzyme for JA-regulated the responses of plant. Transgenic *Arabidopsis* overexpressing *JMT* provides clear defense against the virulent fungus *Botrytis cinere*a (Seo et al., 2001). Additionally, GAMT regulates seed germination (Varbanova et al., 2007). In the current study, recombinant GhJMT1 and GhJMT2 were the critical enzymes which catalyzed the methylation of JA. There are about 59 potential substrates for individual SABATH, consequently, most of SABATH do not catalyze a single substrate (Yang et al., 2006; Huang et al., 2011). For instance, JMT in *Arabidopsis* has 100% relative catalytic activity against JA, whereas only 8% against dihydrojasmonic acid (Seo et al., 2001). O-methyltransferases cLEI3O14 in tomato is most active with JA (relative activity 100%), with methylated benzoic acid (62%) and SA (8%) less efficiently (Tieman et al., 2010). However, recombinan GhJMT1 and GhJMT2 only catalyzed JA to MeJA. There may be other potential substrates of GhJMT1 and GhJMT2 in cotton.

Plant defense may be motivated by exogenous additions of synthetic elicitors such as MeJA or JA (Zhou et al., 2014; Williams et al., 2017). Our qPCR results also showed that the transcript abundance of *GhJMT1* and *GhJMT2* were significantly up-regulated in cotton tissues after MeJA induction. Furthermore, MeJA treatment induces the emission of linalool, β-ocimene, (*Z*)-3-hexenyl acetate, DMNT and other plant volatiles with various important defensive functions. According to the report, emission of monoterpenes and sesquiterpenes in tomato induced by MeJA treatment are significantly higher than those in controls (Farag and Paré, 2002; Baldwin et al., 2006). However, in this study, only the emssion of β-myrcene was down-regulated in MeJA treated cotton plants. This may be caused by the metabolic-synthetic trade-off in cotton.

HIPVs are always thought to function as a direct and indirect form of plant defense by repelling herbivores and recruiting natural enemies (Heil, 2008). Some monoterpenes, such as α-pinene and β-phellandrene, have been reported to attract the generalist predators *Macrolophus pygmaeus* of tomato leaf miner *Tuta absoluta* (De-Backer et al., 2017). The transgenic cotton lines with increased (*E*)-β-caryophyllene emissions not only reduce the herbivorous pests, *Apolygus lucorum*, *Aphis gossypii* and *Helicoverpa armigera*, but also attract two natural enemies, *Peristenus spretus* and *Aphidius gifuensis* (Zhang et al., 2019). In the current study, parasitoid wasp *M. mediator* showed siginificantly electrophysiological responses to MeJA, β-ocimene, *(Z)*-3-hexenyl acetate and linalool suggesting a vital role of MeJA and its induced products in cotton plant defense. Therefore, we propose that GhJMT1 and s GhJMT2 are important methyltransferases in synthesis of MeJA in cotton plants.

## Data availability statement

The original contributions presented in the study are included in the article/supplementary material. Further inquiries can be directed to the corresponding author.

## Ethics statement

The studies involving animals were reviewed and approved by Experimental Animal Welfare and Ethical Committee of Institute of Plant Protection, Chinese Academy of Agricultural Sciences.

## Author contributions

YZ and XH conceived and designed the experiments. DT, WJ, DZ and BL performed the experiments and analyzed the data. DT, WJ, BL, JK, and KHD refined the manuscript. All authors read and approved the final manuscript.
